# Lung cancer treatment and mortality for Aboriginal people in New South Wales, Australia: results from a population-based record linkage study and medical record audit

**DOI:** 10.1186/s12885-016-2322-1

**Published:** 2016-04-25

**Authors:** Alison Gibberd, Rajah Supramaniam, Anthony Dillon, Bruce K. Armstrong, Dianne L. O’Connell

**Affiliations:** School of Public Health, University of Sydney, Sydney, Australia; Cancer Research Division, Cancer Council NSW, Sydney, Australia; Institute for Positive Psychology and Education, Australian Catholic University, Sydney, Australia; School of Medicine and Public Health, University of Newcastle, Newcastle, Australia

**Keywords:** Lung cancer, Patterns of care, Aboriginal people, Cancer survival, Australia/epidemiology

## Abstract

**Background:**

The aim of this study was to compare surgical treatment received by Aboriginal and non-Aboriginal people with non-small cell lung cancer (NSCLC) in New South Wales (NSW), Australia and to examine whether patient and disease characteristics are associated with any disparities found. An additional objective was to describe the adjuvant treatments received by Aboriginal people diagnosed with NSCLC in NSW. Finally, we compared the risk of death from NSCLC for Aboriginal and non-Aboriginal people.

**Methods:**

We used logistic regression and competing risks regression to analyse population-based cancer registry records for people diagnosed with NSCLC in NSW, 2001–2007, linked to hospital inpatient episodes and deaths. We also analysed treatment patterns from a medical record audit for 170 Aboriginal people diagnosed with NSCLC in NSW, 2000–2010.

**Results:**

Of 20,154 people diagnosed with primary lung cancer, 341 (1.7 %) were Aboriginal. Larger proportions of Aboriginal people were younger, female, living outside major cities or in areas of greater socioeconomic disadvantage, smoking at the time of diagnosis and had comorbidities. Although Aboriginal people were, on average, younger at diagnosis with non-metastatic NSCLC than non-Aboriginal people, only 30.8 % of Aboriginal people received surgery, compared with 39.5 % of non-Aboriginal people. Further, Aboriginal people who were not receiving surgery, at the time of diagnosis, were more likely to be younger, live in major cities and have no comorbidities. The observed risk of death from NSCLC 5 years after diagnosis was higher for 266 Aboriginal people (83.3 % 95 % CI 77.5–87.7) than for 15,491 non-Aboriginal people (77.6 % 95 % CI 76.9–78.3) and the adjusted subhazard ratio was 1.32 (95 % CI 1.14–1.52). From the medical record audit, 29 % of Aboriginal people with NSCLC had potentially curative treatment, 45 % had palliative radiotherapy/chemotherapy and 26 % had no active treatment.

**Conclusions:**

There are disparities in NSCLC surgical treatment and mortality for Aboriginal people compared with non-Aboriginal people in NSW. It is imperative that Aboriginal people are offered active lung cancer treatment, particularly those who are younger and without comorbidities and are therefore most likely to benefit, and are provided with assistance to access it if required.

## Background

Lung cancer is the most common cause of cancer death for both the Australian Aboriginal and non-Aboriginal populations [1]. In New South Wales (NSW) the 5-year lung cancer-specific survival for Aboriginal people has been reported to be approximately half that of non-Aboriginal people [2]. The reasons for this difference in survival are complex and have not yet been explored in NSW, although a study from Queensland [3], another state in Australia, attributed most of the difference to disparities in the medical treatment received by Aboriginal and non-Aboriginal people. This study found that, after adjusting for a range of disease and patient characteristics, the probability of Aboriginal people receiving active treatment at any stage of the illness was 35 % lower than for non-Aboriginal people [3]. Similarly, a Western Australian study [4] found that the adjusted odds of receiving surgical treatment were 37 % lower for Aboriginal people than non-Aboriginal people diagnosed with lung cancer.

Surgical resection is the most effective treatment for non-metastatic non-small cell lung cancer (NSCLC), as well as for highly selected cases with a single site of metastases [5, 6]. However, the feasibility of surgery depends on the extent and location of the disease, and the ability of the patient to tolerate the procedure [7]. When surgical resection is not indicated for NSCLC, radiotherapy, chemotherapy and/or palliative management are recommended [5]. The optimal mix of treatments is determined by disease and patient characteristics, including spread of disease, comorbidities and age [5]. It is possible that differences in these factors, as well as barriers to treatment access, lead to differences in the treatment of, and mortality from, lung cancer for Aboriginal and non-Aboriginal people.

To date, no studies of NSCLC treatment for Aboriginal people have been conducted in NSW, which is the most populous state in Australia (approximately 7 million people) and has an estimated 29 % of the total Australian Aboriginal population of approximately 148,000 people [8]. Aboriginal people comprise approximately 2 % of the NSW population and, nationally, have a median age of 21 years compared with a median age for non-Aboriginal people of 37 years [8]. Compared with Queensland and Western Australia, Aboriginal people in NSW are much more likely to live in major cities and inner regional areas [8], and therefore may have better access to specialist lung cancer treatment centres.

We use the descriptor ‘Aboriginal people’ throughout this paper to refer to the original people of Australia and their descendants, as endorsed by the Aboriginal Health and Medical Research Council in NSW and NSW Health [9].

The aim of this study was to compare surgical treatment for NSW Aboriginal and non-Aboriginal people diagnosed with non-metastatic NSCLC, and to examine the degree to which differences in patient and disease characteristics are associated with any disparities found. An additional objective was to describe radiotherapy and chemotherapy treatment for Aboriginal people diagnosed with NSCLC in NSW. Finally, we compared Aboriginal and non-Aboriginal people’s risk of death from NSCLC.

## Methods

The methods used here have been described previously [10–12], and, briefly, involve the analysis of two different linked datasets. The first dataset (“NSW population data”) contained 21,127 incident lung cancer cases for 2001–2007 from the NSW Central Cancer Registry (CCR), linked to hospital episode records and death records. The second dataset (“Patterns of Care data”) comprised data from a medical records audit linked to CCR, hospital and death records. Eligible cases were aged 18 years and over, diagnosed with primary lung cancer (ICD-O-3 codes “C33” and “C34” and morphology codes ending in/3), and resident in NSW at diagnosis. The probabilistic linkage of records in the different datasets was carried out by the Centre for Health Record Linkage (CHeReL) using ChoiceMaker software and privacy-preserving methods (ChoiceMaker Technologies Inc., New York, US). The CHeReL reports approximately 0.1 % false positive and less than 0.1 % false negative linkages.

### Data sources

#### NSW population data

All invasive cancers diagnosed in NSW have been required by statute to be notified to the NSW Central Cancer Registry (CCR) since 1972. All inpatient episodes in all public and private hospitals in NSW are documented in and available from the NSW Ministry of Health’s Admitted Patient Data Collection (APDC).

**Fig. 1:**
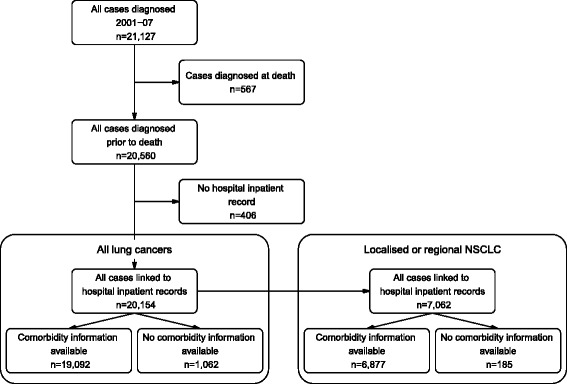
Inclusion and exclusion criteria for the NSW population data of people with lung cancer diagnosed in NSW 2001–2007

As the focus of this study was comparing treatment after diagnosis, we excluded from the analysis 567 people (2.7 %) who were notified to the CCR by death certificate or after autopsy only. The remaining 20,560 people were linked to the APDC for the period 1 July 2000 to 30 June 2009. Death records including Aboriginal status up to 31 December 2007 were obtained from the Australian Bureau of Statistics (ABS). After excluding people with no matching APDC record (406, 1.9 %) as their Aboriginal status was unknown and they may have been treated in hospitals outside NSW [13] 20,154 people were included in the analysis (Fig. 1).

In this analysis, a person was determined to be Aboriginal if they were listed as Aboriginal and/or Torres Strait Islander in any of their matching APDC or ABS records. We have not reported data separately for Torres Strait Islander people as there were very few identified in the source datasets.

Lung cancers were grouped by histological type as NSCLC, small cell lung cancer (SCLC) and “other and unspecified”, similar to the groupings used by the Australian Institute of Health and Welfare [14]. NSCLC included squamous cell carcinoma, adenocarcinoma, large cell carcinoma and the group defined by the Australian Institute of Health and Welfare as “other specified carcinoma”.

Surgical treatment for localised and regional (“non-metastatic”) NSCLC was identified from the procedure codes listed in the APDC. Surgical treatment was defined as pneumonectomy, lobectomy, lung resection or resection of endotracheal tumour. Pleurodesis was not included as the main intent of this procedure is palliative. We restricted our analysis to surgical treatment because radiotherapy and chemotherapy, largely administered in outpatient services, are not routinely recorded in the APDC [13].

Age at diagnosis, sex, local government area (LGA) of residence at time of diagnosis, month and year of diagnosis, spread of disease at diagnosis and histology were obtained from the CCR. Spread of disease at diagnosis was reported by the CCR in four categories: localised (the tumour was contained within the organ in which it originated), regional (the tumour had spread to surrounding organs, adjacent tissue and/or nearby lymph nodes), distant (metastatic disease) and unknown [15]. We could not assess differences between Aboriginal and non-Aboriginal people in the use of Positron Emission Tomography (PET) for cancer staging as we only had inpatient records and PET scans can be done on an outpatient basis.

Each person was allocated to one of three categories of geographic remoteness using the ARIA+ (Accessibility/Remoteness Index for Australia) [16] value for their LGA of residence. The ARIA+ index is calculated using road distances of a LGA to the nearest population centres or ‘service centres’. The service centres are categorised into major cities, inner regional and rural (which included outer regional, remote and very remote) based on population size. Quintiles of socioeconomic disadvantage were obtained by mapping their LGA of residence to the ABS Socio-Economic Indexes for Areas (SEIFA) Index of Relative Socio-Economic Advantage and Disadvantage [17, 18].

Information about comorbidities was obtained from the APDC diagnosis codes, which include the primary reason for hospitalisation and additional comorbidities [19]. The presence of non-cancer comorbidities included in the Charlson Comorbidity Index [20] was obtained from hospital admission records from 12 months prior to diagnosis to 6 months after diagnosis. Those people who were not admitted to a NSW hospital during this 18 month period were excluded from analyses of factors related to receiving treatment (Fig. 1).

Smoking status was obtained from the APDC diagnosis codes. There is no code for non-smokers and it is not mandatory to record smoking status in the APDC. “Current smokers” were those who had a record of being a current smoker after diagnosis. “Former smokers” were those whose last smoking-related diagnosis prior to their cancer diagnosis was former smoker. “Ever smokers” were people with a record of current and/or former smoking, but it was not possible to determine if they were current smokers when they were diagnosed with cancer. “Never smokers” were defined as those who did not have any diagnosis of current or former smoker and were admitted at least once to a NSW hospital that was considered to record smoking status reliably, specifically at least 20 % of admissions had a smoking-related record. For the remainder, smoking status was coded as unknown.

#### Patterns of Care (POC) data

The Patterns of Care data were obtained through a medical records audit of a sample of Aboriginal people resident in NSW diagnosed with any invasive cancer in 2000–2011. Data were collected from 23 public hospitals and three Clinical Cancer Registries in NSW. The hospitals and registries were chosen based on size, recording of Aboriginal status, ability to extract electronic patient lists and the availability of a local Principal Investigator. Field officers confirmed Aboriginal status and extracted diagnosis and treatment information from paper and electronic medical records. In total, data were collected for 1304 Aboriginal people, of whom 219 were diagnosed with lung cancer in 2000–2010.

We collected disease and treatment information using a form largely based on a form developed for a previous study [21]. The data collection form used in this study was reviewed by three oncologists to ensure that it covered all current forms of treatment. Data on disease characteristics included topography, histology, lymph node involvement and evidence of distant metastases. Spread of disease was categorized into three groups: non-metastatic, metastatic and unknown. Information on surgery, radiotherapy and chemotherapy included the timing of treatment, the intent of treatment (curative or palliative), and reasons for no treatment. Stereotactic radiotherapy became available in NSW after 2010 and so was not part of the standard treatment for NSCLC during the study period.

Records in the POC data were linked to the APDC for July 2000 to June 2009, the NSW Registry of Births, Deaths and Marriages death records for January 2000 to June 2010, and the CCR for 2000 to 2008 by the CHeReL. Histological type, place of residence, socioeconomic disadvantage and comorbidities were assigned in the same way as for the NSW population data. When information about treatment was missing in the POC data, but present in the APDC, details from the APDC were used to supplement the POC data.

#### Statistical analysis

Differences between Aboriginal and non-Aboriginal people with lung cancer were tested using Pearson’s chi-squared test. Tests of differences between Aboriginal people in the NSW population data and the POC data were not conducted, due to the overlap in the two datasets.

Logistic regression models were used to compare the odds of having surgical treatment for non-metastatic NSCLC for Aboriginal and non-Aboriginal people in the NSW population data. All models included Aboriginal status as an explanatory variable and the full model also contained: sex, age group, spread of disease at diagnosis, year of diagnosis, comorbidities, socioeconomic disadvantage quintiles and place of residence. Finally, smoking status was added to this model to investigate the additional effect on the odds of surgical treatment for Aboriginal compared to non-Aboriginal people.

Differences in the relationship between Aboriginal status and surgery across strata defined by the other covariates were tested by adding interaction terms to the full logistic regression model, with some strata collapsed, as shown in Table 3 [22]. The difference in the time from diagnosis to surgery for those who had surgery was tested using the Mann–Whitney test.

The risk of death from NSCLC was analysed using competing risks regression [23, 24]. Follow-up was censored at 31 December 2008 for all surviving people, with non-lung cancer deaths treated as the competing risk. The main factor of interest was Aboriginal status. Sex, age group, spread of disease at diagnosis, year of diagnosis, surgical treatment, comorbidities, socioeconomic disadvantage quintiles, place of residence and smoking status were also included in the full regression model. We obtained the sub-distribution hazard ratios (SHRs) for each factor in the full model.

All analyses were performed using SAS software (release 9.3; SAS Institute Inc, Cary, North Carolina), R 3.1.0 [25] and Stata/IC 13.1 (StataCorp).

#### Ethical approval

The study using the NSW population data and the linkage of the Patterns of Care data to NSW health datasets were approved by the NSW Population and Health Services Research Ethics Committee and the Human Research Ethics Committee of the Aboriginal Health and Medical Research Council. Data collection for the Patterns of Care study was approved by the ethics committees of Royal Prince Alfred Hospital and the Aboriginal Health and Medical Research Council. Local Regional Governance Offices granted Site Specific Approval for data collection in participating hospitals and Clinical Cancer Registries. Seeking individual patient consent was determined to be impracticable by the lead ethics committees given the nature of the disease and the retrospective study methods that have been used.

## Results

### NSW population data

Of the 20,154 people with lung cancer diagnosed in NSW in 2001–2007, 341 (1.7 %) were identified as Aboriginal (Table 1). Compared to the non-Aboriginal people, larger proportions of Aboriginal people were female (44 % versus 37 %) or under the age of 60 years at diagnosis (35 % versus 18 %) (Table 1). Aboriginal people were more likely to live outside major cities and in more socioeconomically disadvantaged areas, and were also more likely to be smoking around the time of diagnosis, and have comorbid diabetes or chronic pulmonary disease. Spread of disease at diagnosis was similar for Aboriginal and non-Aboriginal people. SCLC was more common for Aboriginal people, but the difference was not statistically significant.

**Table 1:** Demographic and disease characteristics of Aboriginal and non-Aboriginal people diagnosed with lung cancer in NSW

	NSW population data, diagnosed 2001–2007					Patterns of Care (POC) data, diagnosed 2001–2010	
	Non-Aboriginal		Aboriginal			Aboriginal	
	n	%	n	%	*p*-value^a^	n	%
All people	19,813		341			219	
Sex							
Male	12,540	63	191	56	0.006	122	56
Female	7273	37	150	44		97	44
Age at diagnosis (years)					<0.001		
18–49	930	5	39	11		31	14
50–59	2609	13	79	23		65	30
60–69	5292	27	110	32		71	32
70–79	7117	36	87	26		45	21
80+	3865	20	26	8		7	3
Spread of disease (from CCR)^b^					0.586		
Localised	4602	23	79	23		40	22
Regional	3352	17	67	20		42	23
Distant	7281	37	118	35		67	37
Unknown	4578	23	77	23		30	17
Place of residence at diagnosis^b^					<0.001		
Major cities	13,687	69	125	37		77	43
Inner regional	4637	23	122	36		60	34
Rural^c^	1489	8	94	28		42	23
Comorbidities^d^							
Diabetes	2735	15	67	20	0.004	46	27
Cardiovascular disease	3999	21	85	26	0.055	35	21
Chronic pulmonary disease	5381	29	132	40	<0.001	35	38
Renal disease	899	5	23	7	0.070	10	6
Any other comorbidities	2380	13	39	12	0.624	27	16
Socioeconomic disadvantage quintile^b^				<0.001			
Least disadvantaged	3031	15	22	6		12	7
Second least disadvantaged	4041	20	31	9		15	8
Third least advantaged	3375	17	44	13		36	20
Second most disadvantaged	4476	23	74	22		33	18
Most disadvantaged	4890	25	170	50		83	46
Smoking status					<0.001		
Current smoker	5582	28	143	42		101	46
Ever smoker^e^	5934	30	99	29		61	28
Former smoker	4237	21	56	16		23	11
Never smoker	3623	18	35	10		22	10
Unknown	437	2	8	2		12	5
Histological type (from CCR)^b^					0.059		
Non-small cell	16,369	83	273	80		142	79
Small cell	2533	13	57	17		34	19
Other and unspecified	911	5	11	3		3	2
Method of diagnosis (from CCR)^b^					0.58		
Histopathology	14,205	72	251	74		139	78
Cytology	2832	14	42	12		17	10
Clinical/Imaging/Biochemical	2776	14	48	14		23	13

*CCR* Central Cancer Registry

^a^ *p*-values are from Pearson's χ^2^ test comparing frequencies in Aboriginal and non-Aboriginal people in the NSW population data only

^b^For the POC data, only 179 people who linked to the CCR were included

^c^Rural includes outer regional, remote and very remote

^d^People who were not admitted to a NSW hospital from 12 months prior to 6 months after diagnosis were excluded as information on comorbidities was not available. In the NSW population data, 331 Aboriginal and 18,761 non-Aboriginal people were included. In the POC data, 170 Aboriginal people were included

^e^Current or former smoking status at the time of diagnosis could not be determined

### Surgical treatment for non-metastatic NSCLC

When we restricted the analysis to people diagnosed with non-metastatic NSCLC, 30.8 % of the 120 Aboriginal people received surgery, compared with 39.5 % of non-Aboriginal people. The median time between diagnosis and surgery was similar for Aboriginal and non-Aboriginal people with non-metastatic NSCLC (24 days for Aboriginal and 20 days for non-Aboriginal people, *p* = 0.86). The types of surgical treatment received were similar, with 57 % of Aboriginal people and 58 % of non-Aboriginal people having lobectomies. The age-adjusted odds of having surgery were 46 % lower for Aboriginal than non-Aboriginal people (OR 0.54, 95 % CI 0.36–0.80). After also adjusting for sex, year of diagnosis, spread of disease, place of residence, comorbidities and socioeconomic disadvantage, the difference was reduced (OR 0.70, 95 % CI 0.46–1.05) and no longer statistically significant (Table 2). The proportions who were never smokers in Aboriginal and non-Aboriginal people were 10 % and 18 % respectively (Table 1). The addition of smoking status had little effect on the odds ratio for surgery for Aboriginal compared with non-Aboriginal people (OR 0.68, 95 % CI 0.46–1.03).

**Table 2:** Odds ratios for surgical treatment^a^ for people with non-metastatic non-small cell lung cancer in NSW 2001–2007^b^

	Odds ratio^c^ (95 % CI)	*p*-value
Aboriginal	0.70 (0.46–1.05)	0.084
Sex		0.042
Male	1.00	
Female	1.12 (1.00–1.24)	
Age at diagnosis (years)		<0.001
18–49	1.00	
50–59	0.89 (0.68–1.15)	
60–69	0.90 (0.70–1.14)	
70–79	0.58 (0.46–0.74)	
80+	0.20 (0.15–0.27)	
Spread of disease		<0.001
Localised	1.00	
Regional	0.81 (0.73–0.90)	
Year of diagnosis	1.04 (1.02–1.07)	0.001
Place of residence at diagnosis		<0.001
Major cities	1.00	
Inner regional	0.69 (0.60–0.80)	
Rural^d^	0.66 (0.51–0.83)	
Comorbidities^e^		
Chronic pulmonary disease	1.04 (0.93–1.16)	0.526
Diabetes	1.08 (0.93–1.25)	0.300
Cardiovascular disease	0.71 (0.62–0.82)	<0.001
Renal disease	0.74 (0.56–0.97)	0.029
Other comorbidities	0.60 (0.49–0.72)	<0.001
Socioeconomic disadvantage quintile		<0.001
Least disadvantaged	1.00	
Second least disadvantaged	0.86 (0.73–1.02)	
Third least disadvantaged	0.73 (0.61–0.87)	
Second most disadvantaged	0.85 (0.72–1.01)	
Most disadvantaged	0.63 (0.51–0.76)	

*CI* confidence interval

^a^Surgical treatment includes pneumonectomy, lobectomy, lung resection or resection of endotracheal tumour

^b^There were 120 Aboriginal and 6757 non-Aboriginal people in this analysis

^c^Odds ratio adjusted for all other variables in the table

^d^Rural includes outer regional, remote and very remote

^e^Presence vs absence of each comorbidity

Overall in NSW, women, younger people, those with localised spread of disease, those living in major cities and areas with less socioeconomic disadvantage, and those without cardiovascular disease, renal disease or other comorbidities were more likely to receive surgery for their non-metastatic NSCLC (Table 2). The interaction between Aboriginal status and comorbidities was statistically significant (*p* = 0.018), with Aboriginal people with no comorbidities being approximately half as likely to have surgery as similar non-Aboriginal people (22 % versus 43 %), while there was no difference in proportions for those with at least one comorbidity (35 % versus 36 %) (Table 3). While this was the only statistically significant interaction, the proportions of Aboriginal people having surgery were consistently similar or lower compared to non-Aboriginal people across all categories of all covariates. For example, 51 % of non-Aboriginal people under the age of 60 years had surgery, compared to 37 % of older non-Aboriginal people. By contrast, for Aboriginal people the proportions having surgery were similar in both age groups (33 % for those under the age of 60 years and 30 % for older people).

**Table 3:** Surgical treatment within one year following diagnosis of non-metastatic non-small cell lung cancer in NSW 2001–2007^a^

	Aboriginal	Non-Aboriginal	
	Had surgery n/N (%)	Had surgery n/N (%)	*p*-value^b^
All people	37/120 (30)	2666/6757 (39)	-
Sex			0.972
Male	21/72 (29)	1637/4335 (38)	
Female	16/48 (33)	1029/2422 (42)	
Age at diagnosis (years)			0.367
18–59	13/40 (33)	613/1210 (51)	
60+	24/80 (30)	2053/5547 (37)	
Spread of disease			0.831
Localised	20/64 (31)	1584/3928 (40)	
Regional	17/56 (30)	1082/2829 (38)	
Place of residence			0.641
Major cities	13/39 (33)	2044/4760 (43)	
Inner regional	13/44 (30)	480/1498 (32)	
Rural^c^	11/37 (30)	142/499 (28)	
Comorbidities^d^			0.018
No comorbidities	9/41 (22)	1390/3196 (43)	
At least one comorbidity	28/79 (35)	1276/3561 (36)	
Socioeconomic disadvantage			0.245
Least and second least disadvantaged	5/15 (33)	1095/2477 (44)	
Third least disadvantaged	3/16 (19)	464/1168 (40)	
Second most and most disadvantaged	29/89 (33)	1107/3112 (36)	

^a^There were 120 Aboriginal and 6757 non-Aboriginal people in this analysis

^b^For interaction term in logistic regression containing all variables shown in this table

^c^Rural includes outer regional, remote and very remote

^d^Non-cancer comorbidities included in the Charlson Comorbidity Index

### Risk of death from NSCLC

**Fig. 2:**
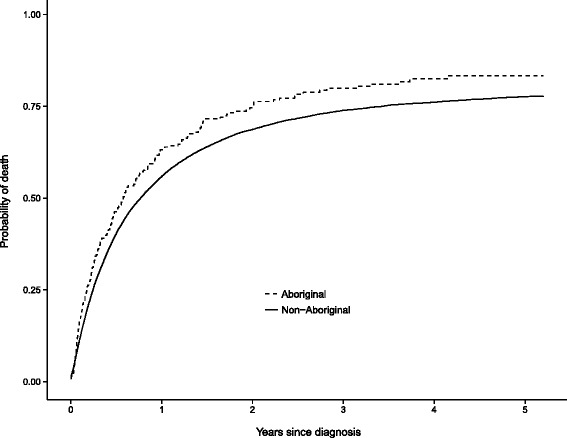
Cumulative risk of death from non-small cell lung cancer for Aboriginal and non-Aboriginal people in NSW, 2001–2007 (*n* = 15,757)

The observed risk of death from NSCLC 5 years after diagnosis was higher for 266 Aboriginal people (83.3 % 95 % CI 77.5–87.7) than for 15,491 non-Aboriginal people (77.6 % 95 % CI 76.9–78.3) (Fig. 2). After adjusting for differences in sex, age at diagnosis, year of diagnosis, spread of disease, place of residence, comorbidities, socioeconomic disadvantage, smoking status and surgical treatment, Aboriginal people with NSCLC had a greater risk of death 5 years after diagnosis compared to non-Aboriginal people (Adjusted SHR 1.32 95 % CI 1.14–1.52). Sex, age at diagnosis, year of diagnosis, spread of disease at diagnosis, having surgical treatment, chronic pulmonary disease, other comorbid conditions, and socioeconomic disadvantage were also significantly associated with the increased risk of death from NSCLC for NSW people (Table 4).

**Table 4:** Competing risks regression model for risk of death from non-small cell lung cancer NSW 2001–2007^a^

Variable name	Subhazard ratio^b^ (95 % CI)	p-value
Aboriginal	1.32 (1.14–1.52)	<0.001
Sex		0.001
Male	1.00	
Female	1.07 (1.03–1.11)	
Age at diagnosis		<0.001
18–50	1.00	
50–59	1.22 (1.11–1.35)	
60–69	1.32 (1.20–1.45)	
70–79	1.51 (1.37–1.66)	
> =80	1.89 (1.71–2.09)	
Spread of disease		<0.001
Localised	1.00	
Regional	1.46 (1.37–1.55)	
Distant	2.58 (2.44–2.73)	
Unknown	1.13 (1.06–1.20)	
Year of diagnosis	0.97 (0.96–0.98)	<0.001
Surgical treatment^c^		<0.001
Did not have surgical treatment	1.00	
Had surgical treatment	0.27 (0.25–0.28)	
Place of residence at diagnosis		0.889
Major cities	1.00	
Inner regional	0.99 (0.94–1.05)	
Rural^d^	1.01 (0.93–1.10)	
Comorbidities^e^		
Diabetes	1.00 (0.94–1.06)	0.976
Cardiovascular disease	0.98 (0.93–1.04)	0.575
Chronic pulmonary disease	1.18 (1.12–1.23)	<0.001
Renal disease	0.94 (0.84–1.05)	0.280
Other comorbidities	1.14 (1.07–1.22)	<0.001
Socioeconomic disadvantage quintile		<0.001
Least disadvantaged	1.00	
Second least disadvantaged	1.04 (0.98–1.11)	
Third least disadvantaged	1.17 (1.09–1.25)	
Second most disadvantaged	1.11 (1.04–1.19)	
Most disadvantaged	1.12 (1.04–1.20)	
Smoking status		0.149
Never smoker	1.00	
Current smoker	1.03 (0.98–1.08)	
Ever smoker	1.06 (1.00–1.12)	
Ex smoker	0.99 (0.93–1.05)	
Unknown	1.01 (0.87–1.17)	

*CI* confidence interval

^a^There were 266 Aboriginal and 15,491 non-Aboriginal people in this analysis

^b^Subhazard ratio adjusted for all other variables in the table

^c^Surgical treatment included pneumonectomy, lobectomy, lung resection or resection of endotracheal tumour

^d^Rural includes outer regional, remote and very remote

^e^Presence vs absence of each comorbidity

### Patterns of care data

Medical records for a sample of 219 Aboriginal people with lung cancer were reviewed. Records were extracted after death for 172 people. For the remaining 47 people, follow up was between 5 and 81 months, with a median follow up of 17 months. Of the 219 people in the sample, 174 had NSCLC, although four of these had incomplete treatment information.

While we could not make formal statistical comparisons, the characteristics of the Aboriginal people in the POC data were similar to those in the NSW population data, except that those in the POC data were slightly younger, less likely to have unknown spread of disease at diagnosis and were slightly more likely to live in a major city than the Aboriginal people in the NSW population data (Table 1).

### Treatment received for NSCLC

Potentially curative treatment was received by half (47/94) of people with non-metastatic NSCLC and for 34/47 (72 %) of these the primary treatment was surgery. By contrast, only 2/71 (3 %) people with metastatic NSCLC received potentially curative treatment, while 51/71 (72 %) received palliative radiotherapy/chemotherapy. Of the 21 people with non-metastatic NSCLC who did not have treatment, eight died within 30 days of diagnosis. Of the remaining 13, the reasons for not being treated were: had comorbidities or they were considered too old to gain any benefit (6); patient choice (2); the tumour was unresectable (3); and no reason provided (2) (Table 5).

**Table 5:** Treatment received within one year following diagnosis by 170 Aboriginal people with non-small cell lung cancer in NSW 2000–2010

		Surgery^a^ +/− radiotherapy/chemotherapy	Potentially curative radiotherapy/chemotherapy	Palliative radiotherapy/chemotherapy	No treatment
Spread of disease	N	Number (%)	Number (%)	Number (%)	Number (%)
Non-metastatic	94	34 (36)	13 (14)	26 (28)	21 (22)
Metastatic	71	1 (1)	1 (1)	51 (72)	18 (25)
Unknown spread	5	-	-	-	5 (100)
Total	170	35 (21)	14 (8)	77 (45)	44 (26)

^a^Surgical treatment included pneumonectomy, lobectomy, lung resection or resection of endotracheal tumour

## Discussion

### NSW population data

For Aboriginal people in NSW with non-metastatic NSCLC, the adjusted odds of having surgical treatment were 30 % lower compared with non-Aboriginal people. This finding was broadly similar to the findings from a Queensland study, where the probability of surgery for Aboriginal people compared to non-Aboriginal people, matched on age, sex and place of residence, was 61 % lower [3] and in Western Australia (where the odds were 37 % lower) [4]. However these studies included all cases of lung cancer, not just non-small cell lung cancer. NSW Aboriginal people also had a higher risk of death from their non-metastatic NSCLC compared with non-Aboriginal people after adjusting for differences in sex, age at diagnosis, year of diagnosis, spread of disease, place of residence, comorbidities, socioeconomic disadvantage, smoking status and surgical treatment.

In NSW, non-Aboriginal people with non-metastatic NSCLC who were younger at diagnosis, without comorbidities or living in major cities were more likely to have surgical treatment. However, this was not true for Aboriginal people. Younger Aboriginal people had only slightly higher rates of surgery than older Aboriginal people, and Aboriginal people living in major cities had similar rates of surgery as those living in inner regional and rural areas, despite their geographical proximity to major hospitals and specialists. Counter-intuitively, Aboriginal people with comorbidities had a higher rate of surgery than those without comorbidities. The opposite was true for non-Aboriginal people. This last result is similar to that found in the Northern Territory [26], where the authors suggested that this may be due to lung cancer being incidentally detected in people under medical surveillance for other lung conditions. However, given the small number of Aboriginal people in our study with non-metastatic NSCLC, and the lack of information on long term comorbidities, no firm conclusions can be drawn. Most healthcare in Australia is paid for by Medicare, a government run universal health care system, through supply of care without charge in public hospitals and subsidised medical services and pharmaceuticals. Some services may require a co-payment when the fee charged is in excess of the subsidy and additional costs such as transport to services, parking and accommodation (except in hospital) are not reimbursed by Medicare. In addition, many private hospitals provide the infrastructure needed for cancer care and private health insurance is available to cover at least a part of the cost of private hospital care. By arrangement in some regional areas private cancer services provide care for public patients free of charge when no public services are available. Specialised cancer services in NSW are largely located in major cities or inner regional areas. Therefore the lower proportion with private health insurance amongst Aboriginal people [27] may have also contributed to the lower surgical treatment rate, as it has been previously reported [28] that NSW residents with private health insurance (or with coverage by the Department of Veterans’ Affairs) with localised NSCLC had twice the odds of receiving a resection compared to people who were uninsured.

The observed increased risk of death from NSCLC for Aboriginal people compared to non-Aboriginal people is similar to the patterns we have previously reported for women with breast cancer [10] and for men with prostate cancer in NSW [11], and also similar to results others have reported for lung cancer in Queensland [3] and the Northern Territory [26] and NSW [2]. Similar increased risks have been shown for Maoris in New Zealand [29], and Canadian First Nations [30] and Inuit [31] peoples.

A limitation of the NSW population data is potential misclassification of Aboriginal and non-Aboriginal people in the APDC. However, an audit in 2007–08 found that all 2661 non-Aboriginal patients interviewed were correctly classified as non-Aboriginal in the APDC [32], suggesting that such misclassification is rare. In the same audit, 93 % of people who identified as Aboriginal at interview were recorded as Aboriginal in the APDC [32]. The proportion of Aboriginal people misclassified in our study is likely to be even lower, as we used any recording of Aboriginal status in any linked records to assign a person’s Aboriginal status. If the Aboriginal people who were misclassified received more (or less) treatment than the Aboriginal people who were correctly classified, our results could be biased away from (or to) the null hypothesis of similar patterns of care.

Major strengths of this study are that it was population-based and the first study of treatment of lung cancer for Aboriginal people in NSW. NSW has the largest Aboriginal population and, compared with Queensland and Western Australia, where the two previous studies were conducted, a greater proportion of NSW Aboriginal people lived in areas close to the major hospitals where lung cancer treatment is predominantly provided.

### Patterns of care data

One half of Aboriginal people with non-metastatic NSCLC in the Patterns of Care data received potentially curative treatment in the first 12 months after diagnosis, 28 % had palliative radiotherapy and/or chemotherapy only, and 22 % had no treatment.

Limitations of the Patterns of Care data include the non-random sampling of hospitals from which medical records were extracted, and the exclusion of people who did not attend a hospital following their lung cancer diagnosis. As a result, the participants may not be representative of all Aboriginal people with lung cancer diagnosed in 2000–2010. However, the demographic and disease characteristics of the Aboriginal people in the POC data and the NSW population data were broadly similar, suggesting that the cases in the POC data may indeed be a good representation of Aboriginal people with lung cancer in NSW. Also, because only people who attended hospital after a lung cancer diagnosis were included in the POC data, the proportions who received treatment may be overestimated. However, this bias may be relatively small as the proportion of Aboriginal people with non-metastatic NSCLC receiving surgery was similar in the NSW population data and the POC data (31 % and 36 %).

Cultural, logistical and socio-economic barriers might explain some of this lack of optimal care. For example limited access to transport or childcare may restrict the ability to undergo treatment [33]. Aboriginal people in NSW have been shown to have a lower health literacy in relation to cancer [34] and can perceive a lack of social inclusion [35] with healthcare systems and these may be barriers to them receiving optimal care. Thompson et al. [33] proposed a number of recommendations to increase the access to new cancer services for Aboriginal people including considering public transport and parking facilities, allowing room for families to visit and/or attend appointments and providing childcare facilities. The same authors [36], as well as Davidson et al. [37] in a review, also suggested that addressing cultural needs and beliefs as well as reducing upfront medical, transport and parking costs for Aboriginal people is likely to improve their access to existing cancer services.

## Conclusions

There is a disparity in the surgical treatment of NSCLC between Aboriginal and non-Aboriginal people in NSW. Counter-intuitively this is particularly true for Aboriginal people who were younger, lived in major cities and inner regional areas, or those without comorbidities. However, a reasonable proportion of Aboriginal people received radiotherapy and chemotherapy. It is therefore possible that the disparity in surgical treatment received, particularly for those diagnosed before 60 years of age, those without comorbidities, or those living in urban areas, is the major contributor to the increased risk of death from lung cancer for Aboriginal people. Consequently it is imperative that Aboriginal lung cancer patients who are most likely to benefit from active treatment are offered such treatment, and are provided with assistance to access it if required.

## Availability of data and materials

Data analysed for this paper are not able to be shared on any publicly available repository due to NSW privacy laws. Approvals would be required from the lead ethics committee as well as the data custodians, before any further data could be provided.
